# Complex phylogenetic distribution of a non-canonical genetic code in green algae

**DOI:** 10.1186/1471-2148-10-327

**Published:** 2010-10-26

**Authors:** Ellen Cocquyt, Gillian H Gile, Frederik Leliaert, Heroen Verbruggen, Patrick J Keeling, Olivier De Clerck

**Affiliations:** 1 Phycology Research Group and Center for Molecular Phylogenetics and Evolution, Ghent University, Krijgslaan 281 S8, 9000 Ghent, Belgium; 2 Canadian Institute for Advanced Research, Department of Botany, University of British Columbia, Vancouver, V6 T 1Z4 Canada

## Abstract

**Background:**

A non-canonical nuclear genetic code, in which TAG and TAA have been reassigned from stop codons to glutamine, has evolved independently in several eukaryotic lineages, including the ulvophycean green algal orders Dasycladales and Cladophorales. To study the phylogenetic distribution of the standard and non-canonical genetic codes, we generated sequence data of a representative set of ulvophycean green algae and used a robust green algal phylogeny to evaluate different evolutionary scenarios that may account for the origin of the non-canonical code.

**Results:**

This study demonstrates that the Dasycladales and Cladophorales share this alternative genetic code with the related order Trentepohliales and the genus *Blastophysa*, but not with the Bryopsidales, which is sister to the Dasycladales. This complex phylogenetic distribution whereby all but one representative of a single natural lineage possesses an identical deviant genetic code is unique.

**Conclusions:**

We compare different evolutionary scenarios for the complex phylogenetic distribution of this non-canonical genetic code. A single transition to the non-canonical code followed by a reversal to the canonical code in the Bryopsidales is highly improbable due to the profound genetic changes that coincide with codon reassignment. Multiple independent gains of the non-canonical code, as hypothesized for ciliates, are also unlikely because the same deviant code has evolved in all lineages. Instead we favor a stepwise acquisition model, congruent with the ambiguous intermediate model, whereby the non-canonical code observed in these green algal orders has a single origin. We suggest that the final steps from an ambiguous intermediate situation to a non-canonical code have been completed in the Trentepohliales, Dasycladales, Cladophorales and *Blastophysa *but not in the Bryopsidales. We hypothesize that in the latter lineage an initial stage characterized by translational ambiguity was not followed by final reassignment of both stop codons to glutamine. Instead the standard code was retained by the disappearance of the ambiguously decoding tRNAs from the genome. We correlate the emergence of a non-canonical genetic code in the Ulvophyceae to their multinucleate nature.

## Background

The genetic code, which translates nucleotide triplets into amino acids, is universal in nearly all genetic systems, including viruses, bacteria, archaebacteria, eukaryotic nuclei and organelles [1,2]. However, a small number of eubacterial, eukaryotic nuclear, plastid and mitochondrial genomes have evolved slight variations of the standard or canonical genetic code [3,4]. Most codon reassignments have been traced to changes in tRNAs, either by single nucleotide substitution, base modification, or RNA editing. Two main models have been proposed to explain the evolutionary changes in the genetic code [reviewed in [4-7]]. The "codon capture" model [8] proposes that bias in GC content can eliminate certain codons from the entire genome, after which they can reappear through random genetic drift, and become reassigned ("captured") by mutations of noncognate tRNAs. This mechanism is essentially neutral, that is, codon reassignment is accomplished without the generation of aberrant and non-functional proteins. The "ambiguous intermediate" model is a non-neutral model, which posits that codon reassignment occurs through an intermediate stage where a particular codon is ambiguously decoded by both the cognate tRNA and a tRNA that is mutated at locations other than at the anticodon, after which the mutant tRNA may take over decoding of the ambiguous codon if it is adaptive [9]. The two models are not mutually exclusive and codon reassignments may be driven by a combination of different mechanisms [10,11]. For example, ambiguous intermediate stages may be preceded by strong GC bias [12].

Similar evolutionary mechanisms of gradual codon reassignment have been suggested to apply to reassignment of stop codons to sense codons, in which a mutated tRNA may initially recognize and eventually capture a stop codon from the cognate release factor [5]. Stop codons may be particularly prone to reassignment either because they are less prevalent than sense codons (occurring only once per gene) and therefore cause minimal detrimental effects if they are reassigned, or because changes to release factors can occur rapidly [4,13].

Only five eukaryotic lineages are known to have evolved non-canonical nuclear genetic codes, including ciliates, hexamitid diplomonads, fungi (in the genus *Candida *and many ascomycetes), polymastigid oxymonads, and green algae (in the Dasycladales and Cladophorales). By far the most common variant is the reassignment of the stop codons TAG and TAA (TAR) to glutamine, which has occurred independently in hexamitid diplomonads [14], several ciliates [15,16], polymastigid oxymonads [17,18] and dasycladalean and cladophoralean green algae [19-21]. Interestingly, this TAR→Gln reassignment has never been observed in prokaryotes or organelles.

The aim of the present study is to document the phylogenetic distribution of the non-canonical genetic code in green algae, with emphasis on the ulvophycean relatives of the Dasycladales and Cladophorales. Our approach consists of screening green algal nuclear genes for the presence of non-canonical glutamine codons and interpreting the evolution of the genetic code and glutamine codon usage in a phylogenetic framework. The apparently complex phylogenetic distribution of a non-canonical code in closely related ulvophycean lineages offers a unique opportunity to study the mechanisms of the protein translational machinery that may have led to codon-reassignment.

## Results

The dasycladalean genera *Acetabularia, Batophora *and *Parvocaulis*, and the cladophoralean genera *Cladophora *and *Chaetomorpha *have been shown previously to use the TAR→Gln genetic code [19-21]. We have characterized one or more of 8 different genes from 21 taxa representing the breadth of ulvophycean diversity [this study and [22], see also Additional files 1 and 2]. From these data, we found that the non-canonical glutamine codons appear in nuclear-encoded genes of additional members of the both Dasycladales (genus *Bornetella*) and Cladophorales (genera *Boergesenia, Boodlea*, *Cladophora*, *Dictyosphaeria*, *Phyllodictyon, Siphonocladus, Valonia*), as well as Trentepohliales (genus *Trentepohlia*) and the genus *Blastophysa*, which is currently not assigned to an order. We inferred that an organism uses a non-canonical code if TAR codons were found at highly conserved positions where land plants and other green algae encode glutamine. Only TGA is used as stop codon in EST sequences that we obtained for *Cladophora *(40 S ribosomal protein S9, GQ421515; OEE1, GQ421494). Moreover, other sequences deposited in GenBank from *Cladophora *(GapA, DQ270261; EF-1α, EF551321) and *Acetabularia *(PsbS, BK006014) terminate with TGA codons only. The presence of TGA as the only stop codon for species having TAR at conserved glutamine positions further supports our inference of a non-canonical genetic code.

The presence of the standard code was determined for the genus *Ignatius *and the order Bryopsidales (genera *Caulerpa*, *Bryopsis*) by the presence of only canonical glutamine codons and the use of all three stop codons. The *Ignatius *actin gene has TAG as stop codon, while the β tubulin and HPS90 genes have TAA as stop codons. For *Caulerpa*, both β tubulin and HPS90 genes have TAA as stop codons, so we did not observe the use of TAG for stop. In publicly available sequences from *Bryopsis *all three stop codons are used: TAA in ribonuclease Bm2 gene (AB164318), TAG in lectin precursor and oxygen evolving protein of photosystem II genes (EU410470 and AB293980), and TGA in the bryohealin precursor gene (EU769118). Taken together, this shows that the Bryopsidales use the standard genetic code.

**Figure 1:**
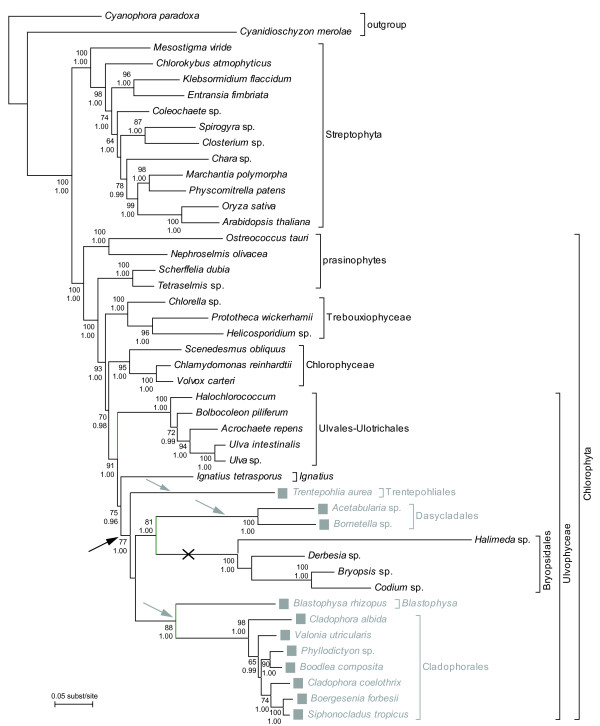
**Phylogenetic distribution of the standard and non-canonical genetic codes in green algae**. The occurrence of a non-canonical genetic code (TAR→Gln) is indicated with gray squares. The taxa with the non-canonical code do not form a monophyletic group Three alternative scenarios can explain this phylogenetic distribution: (1) A single origin of the non-canonical code along the branch leading to the orders Trentepohliales, Dasycladales, Bryopsidales, Cladophorales and the genus *Blastophysa *and a subsequent reversal to the universal code in the Bryopsidales (indicated with black arrow and cross). (2) Three independent gains of the non-canonical code in the Trentepohliales, the Dasycladales and the Cladophorales + *Blastophysa *(indicated with gray arrows). (3) A stepwise process of evolution of the non-canonical code with a single initiation of the process along the branch leading to the orders Trentepohliales, Dasycladales, Bryopsidales, Cladophorales and the genus *Blastophysa*, followed by a completion of the process in all lineages except the Bryopsidales (black arrow combined with gray arrows). The reference phylogeny of the green plant lineage was obtained by maximum likelihood inference of a 25% site stripped dataset containing 7 nuclear genes, SSU nrDNA and plastid genes *rbc*L and *atp*B [22]. Numbers at nodes indicate ML bootstrap values (top) and posterior probabilities (bottom); values below respectively 50 and 0.9 are not shown.

The occurrence of the standard and TAR→Gln code is plotted onto the reference phylogeny in Figure 1. The Streptophyta, prasinophytes, Trebouxiophyceae and Chlorophyceae possess the standard genetic code. Within the class Ulvophyceae, the standard code is found in the orders Ulvales, Ulotrichales, Bryopsidales and the genus *Ignatius *whereas the orders Dasycladales, Cladophorales, Trentepohliales and the genus *Blastophysa *have a non-canonical code. However, the taxa with the TAR→Gln code do not form a monophyletic group (Figure 1).

If both the phylogeny and the distribution of the genetic codes shown in Figure 1 are correct, then more than one gain and/or loss event of the non-canonical code must be postulated. To examine the validity of the phylogeny, we performed AU topology tests to evaluate whether the data rejected a topology in which all taxa with the non-canonical code formed a monophyletic group. Specifically, we found that the topology where Bryopsidales is sister to all Ulvophyceae with the TAR→Gln code is rejected with high significance (ΔlnL = 27.9; p < 0.0001).

**Figure 2:**
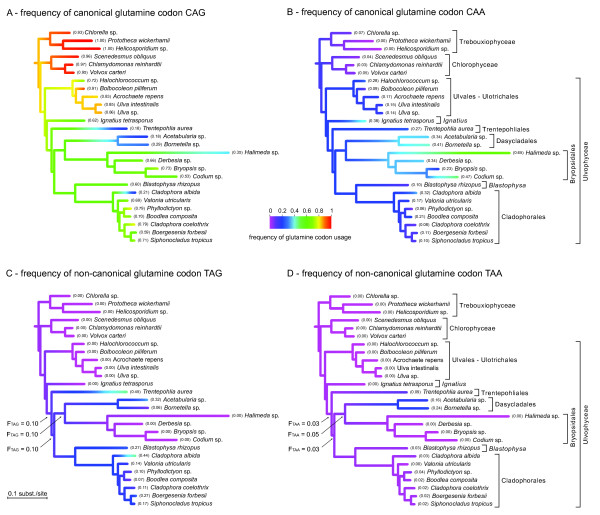
**Estimated ancestral frequencies of glutamine codon usage**. Canonical codon CAG is most commonly used, followed by the other canonical codon, CAA. Among the non-canonical codons, TAG is used more commonly then TAA. The estimate ancestral frequencies of non-canonical codon usage along the nodes of interest are indicated with arrows.

The estimated evolution of glutamine codon usage frequencies is shown in Figure 2 (only the green algal classes Chlorophyceae, Trebouxiophyceae and Ulvophyceae are shown). In general, the canonical codon CAG is most commonly used, followed by the canonical codon CAA. The dominance of the canonical glutamine codons CAG over CAA is also reflected in the non-canonical glutamine codons, TAG being much more common than TAA. The bias towards CAG and TAG is likely a product of the overall bias of these species and/or genes for GC residues, leading to increased use of G in the third codon position. Changes from canonical to non-canonical glutamine codons require a single transition (C → T) at the first codon position.

## Discussion

Our results reveal a broad distribution of a non-canonical nuclear genetic code in the Ulvophyceae, where glutamine is encoded by canonical CAG and CAA codons as well as non-canonical TAG and TAA codons. Surprisingly, we find that taxa with this non-canonical code do not form a monophyletic group according to the seemingly robust phylogeny of the organisms (Figure 1). If the inferred phylogeny is indeed correct, three alternative scenarios can explain the distribution of the code on that tree: (1) a single origin of the non-canonical code along the branch leading to the orders Trentepohliales, Dasycladales, Bryopsidales, Cladophorales and the genus *Blastophysa *and a subsequent reversal to the standard code in the Bryopsidales (Figure 1: indicated with black arrow and cross), (2) three independent gains of the non-canonical code in the Trentepohliales, the Dasycladales and the Cladophorales + *Blastophysa *(Figure 1: indicated with gray arrows), and (3) a stepwise process of evolution of the non-canonical code with a single initiation of the process along the branch leading to the orders Trentepohliales, Dasycladales, Bryopsidales, Cladophorales and the genus *Blastophysa*, followed by a completion of the process in all lineages except the Bryopsidales (Figure 1: black arrow combined with gray arrows). Alternatively, because changes in the genetic code are so rare, the possibility that the reference phylogeny is wrong should not be passed over too easily. More specifically, if one assumes that the current position of the Bryopsidales is wrong and that in reality this group is sister to all the taxa with a non-canonical code, only a single transition to a non-canonical genetic code would have to be invoked. In what follows, we will discuss each of these possibilities in more detail and report on some cytological correlates of the non-canonical genetic code.

### Phylogenetic uncertainty

Our phylogenetic tree is based on the most comprehensive dataset currently available for the Ulvophyceae. It is inferred from a concatenated dataset including seven nuclear genes, SSU nrDNA and two plastid genes using model-based techniques with carefully chosen partitioning strategies and models of sequence evolution and application of a site removal approach to optimize the signal-to-noise ratio [22]. Our tree shows a sister relationship between Dasycladales and Bryopsidales with moderate to high statistical support (BV 81 and PP 1.00). This relationship is concordant with a recently published 74-taxon phylogeny of the green lineage based on SSU nrDNA and two plastid genes [23]. Both phylogenies show major improvements in taxon and gene sampling within the Ulvophyceae compared to previously published phylogenies, which were either based on a single marker, did not include the Bryopsidales, or could not resolve the relationships among the Bryopsidales, Dasycladales and Cladophorales [24-26]. Based on the dataset used to infer our reference tree, the alternative topology in which the Bryopsidales are sister to all taxa with a non-canonical code is significantly less likely than the ML tree as shown by AU tests. As a consequence, we must conclude based on all available information that the ulvophycean taxa with the non-canonical code form a paraphyletic group and one of the more complex evolutionary scenarios for the gain of the non-canonical code has to be invoked.

### Gain-reversal hypothesis

A reversal from the non-canonical to the standard genetic code is unlikely for several reasons, indeed arguably more unlikely than the original change to the non-canonical code. Following a transition to the non-canonical code, TAR glutamine codons would be present in many coding sequences. In order to revert to the canonical code, these codons would all have to revert to canonical CAR codons or they would terminate translation, with obvious detrimental effects. The improbability of this reversal is enforced by the increased frequency of the codons: stop codons appear only once per gene, whereas glutamine is present many times per gene on average. Our ancestral state estimates indicate a non-negligible usage frequency of both non-canonical codons along the ancestral nodes of interest (Figure 2C, D, indicated with arrows). These results must be considered with caution because of the intrinsic limitations of ancestral state estimation [27] and the fact that the non-independence of the evolution of the genetic code and glutamine codon usage cannot be captured by the Brownian motion model. The possibility of a reversal from a non-canonical to a canonical code cannot be ruled out entirely. For example, this could have been possible through an ambiguous intermediate stage in which TAR codons are both recognized by normal glutamine tRNAs and release factors. It is worth mentioning here that gain-reversal scenarios have been demonstrated in a number of cases. For example in arthropod lineage mitochondrial genomes, reassignment of AGG from Ser to Lys has been shown to occur at the base of the arthropods and has been reversed to the normal code several times independently. These reversals have been correlated with mutations in anti-codons of the tRNA-Lys/-Ser and with low abundance of the AGG codon [28]. Nevertheless, the major argument against a reversal from a stop to sense reassignment as observed in the Ulvophyceae is the sudden appearance of many internal stop codons. In addition, it has been suggested that the non-canonical TAR→Gln code is more robust to error than the standard code, further reducing the likelihood of reversals [29]. Taken together, the reversal of a non-canonical code to the standard code appears highly unlikely.

### Multiple independent gains

Several independent acquisitions of non-canonical codes have been reported for ciliates [4,30-32]. Stop codon reassignments are surprisingly frequent in this group of organisms: the same non-canonical TAR→Gln code has evolved several times, another non-canonical code in which TGA codes for tryptophan evolved twice, a non-canonical code that translates TGA to cysteine evolved once and a fourth non-canonical codes which translates TAA into glutamic acid has been reported for three ciliate species. In the present study, the distribution of the non-canonical code in the phylogenetic tree would require three gains: in the Trentepohliales, the Dasycladales and the Cladophorales + *Blastophysa*. Contrary to the situation in ciliates, however, Ulvophyceae only evolved a single type of non-canonical code and they did so in closely related lineages, and even within the ciliates it has been suggested that the reassignment of TAR codons to glutamine in some oligohymenophoran lineages and glutamic acid in others may share a common origin [32]. It seems unparsimonious to suggest that such a rare occurrence as codon reassignment could take place three times independently in three closely related lineages, but the possibility cannot be excluded altogether.

### Stepwise acquisition model

Several studies have suggested that stop codon reassignment is a gradual process requiring changes to tRNA and eukaryotic release factor 1 (eRF1) genes [reviewed in [4,5,10]]. In several eukaryotes, the two glutamine tRNAs recognizing CAG and CAA are also able to read TAR codons by a G·U wobble base pairing at the third anticodon position [33]. Under normal circumstances (standard code), the eukaryotic release factor outcompetes the glutamine tRNAs when it comes to binding TAG and TAA. Mutations in glutamine tRNA copies that allow them to bind TAG and TAA may increase the capacity to translate TAG and TAA to glutamine. This leads to an intermediate step in which both eRF1 and the mutated tRNAs can easily bind to TAR glutamine codons. The ciliate *Tetrahymena thermophila *has three major glutamine tRNAs, one that recognizes the canonical CAR codons, and two supplementary ones that recognize the non-canonical TAR codons. These two supplementary tRNAs were shown to have evolved from the normal glutamine tRNA [16]. A similar situation likely exists in diplomonads [14]. In order to alter the genetic code, mutations are required not only in glutamine tRNAs but also in eRF1 so they no longer recognize TAG and TAA codons. In ciliates it has been shown that eRF1 sequences are highly divergent in domain 1 between species with a canonical and non-canonical code, which might suggest that eRF1 can no longer recognize TAG and TAA codons in the species with a non-canonical code [31,34]. An additional mechanism that constrains the evolvability of the genetic code and therefore represents yet another potential step in the process of codon reassignment is nonsense-mediated mRNA decay (NMD) [35,36]. NMD reduces errors in gene expression by eliminating mRNAs that encode for an incomplete polypeptide due to the presence of stop codons. In the case of a TAR→Gln code this NMD mechanism would have to be altered also in order to prevent degradation of mRNAs containing TAG and TAA codons.

A gradual, stepwise acquisition model of codon reassignment can reconcile the opposed and problematic hypotheses of multiple gains versus a single gain with subsequent loss. For example, the ambiguous intermediate model [5,9] would explain the distribution of the non-canonical code in Ulvophyceae as follows: mutations in the anticodons of canonical glutamine tRNAs occurred once along the branch leading to the orders Trentepohliales, Dasycladales, Bryopsidales, Cladophorales and the genus *Blastophysa *(Figure 1, black arrow). The presence of these mutated tRNAs allowed TAG and TAA codons to be translated to glutamine instead of terminating translation. At this step, the mutated tRNAs compete with eRF1 for the TAA and TAG codons. To complete the transition to the non-canonical code, subsequent mutation of the release factors preventing termination for TAG and TAA codons is required. If one assumes that this step occurred three times independently in the Trentepohliales, Dasycladales and Cladophorales + *Blastophysa *(Figure 1, gray arrows), whereas the mutated tRNAs decreased in importance or went extinct through selection or drift along the branch leading to the Bryopsidales, the distribution of the non-canonical code in the Ulvophyceae would be explained. A detailed comparison of eukaryotic release factors and glutamine tRNAs in the respective clades of the Ulvophyceae is needed to verify this evolutionary scenario.

Alternatively, the observed distribution could also be explained under a stepwise version of the codon capture model [13]. We then assume that the TAR stop codons disappeared from a common ancestor (Figure 1: black arrow) and were subsequently reassigned independently to glutamine codons in the Trentepohliales, Cladophorales + *Blastophysa *and Dasycladales (Figure 1: grey arrows) and reappeared with their old function in the Bryopsidales. Although there is some evidence for different GC usage patterns in Ulvophyceae and earlier-branching Chlorophyta [37], we consider codon capture an unlikely candidate to explain TAR→Gln codon reassignments because a pressure towards either AT or GC across the entire genome cannot explain the extinction of both TAA and TAG codons.

The stepwise acquisition model with ambiguous intermediates is expected to reduce organismal fitness during intermediate stages due to competition between release factors and glutamine tRNAs resulting in aberrant peptides. However, the fact that several eukaryotes have natural nonsense suppressor tRNAs that can translate stop codons, though generally at low efficiency [33], and that these have been maintained over evolutionary time, suggest that readthrough may not be a severe problem and could even increase fitness during periods of environmental instability [5]. Considering that the Ulvophycean orders diversified during the environmentally instable Cryogenian [37-39] and that early-branching Ulvophyceae occur in a variety of habitats including marine, freshwater and terrestrial ecosystems, one could speculate that genetic code ambiguity may have been advantageous during their early evolution.

### Cytological correlates of non-canonical code

In the ciliates, the multiple appearances of alternative codes have been attributed to their nuclear characteristics [40]. Ciliates are unicellular organisms with two nuclei: a small, diploid micronucleus which is not expressed and represents the germ line for DNA exchanges during the sexual process, and a large, polyploid macronucleus, which is actively transcribed and ensures vegetative cell growth, but is not passed on to progeny after sexual conjugation and is replaced by a newly formed macronucleus after a number of rounds of mitotic division. There is therefore a time lag between the occurrence of mutations in the micronucleus and the expression of these mutations in the macronucleus, and this has been postulated to be a contributing factor to why ciliates have evolved alternative genetic codes more frequently [40]. In this context it is worth mentioning that hexamitid diplomonads, for which a single origin of a non-canonical code has been shown, have two semi-independent but similar nuclei per cell [17]. The same genetic code is also used by the uninucleate enteromonads, but enteromonads are known to have evolved from within diplomonads [41], so the code originated in a binucleate ancestor. Several ulvophycean groups are also characterized by multinucleate cells, namely the Dasycladales, Bryopsidales, Cladophorales and *Blastophysa *[22]. However, it should be mentioned that the Trentepohliales, which also uses the non-canonical code, is characterized by uninucleate cells. The Cladophorales and *Blastophysa *are branched filamentous seaweeds consisting of multinucleate cells with a few to thousands of nuclei arranged in non-motile cytoplasmic domains (siphonocladous organization). Members of the Bryopsidales and Dasycladales have a siphonous organization: they consist of a single, giant tubular cell with a single giant nucleus or with numerous nuclei, and complex patterns of cytoplasmic streaming. The presence of multiple nuclei per cell might provide an opportunity to experiment with the genetic code because the cell as an entity might still be able to function normally and each nucleus can potentially be passed to the next generation. Despite the fact that some eukaryotes with a non-canonical code do not feature multinucleate cells and that there are plenty of examples of groups with multinucleate cells that have not evolved alternative codes, this observation suggests that a multinucleate cytology may facilitate codon reassignments.

## Conclusions

We demonstrate that the Dasycladales and Cladophorales share the TAR to glutamine reassignment with the related order Trentepohliales and the genus *Blastophysa*, but not with the Bryopsidales, which is sister to the Dasycladales. We discuss several alternative scenarios for the origin of this complex distribution of the non-canonical code: phylogenetic uncertainty, gain-reversal hypothesis, multiple independent gains and stepwise acquisition model. Considering the robustness of our phylogeny, the profound genetic changes that coincide with codon reassignments and the scarcity of codon reassignments, we conclude that a stepwise acquisition model is the most likely hypothesis. The present study has provided a framework to better understand the evolution of the genetic code. Further insights will be gained by sequencing and analyzing release factors and glutamine tRNAs of taxa using non-canonical codes.

## Methods

### RNA isolation, polymerase chain reaction and sequencing

Total RNA was extracted from 43 taxa representing the major lineages of the Viridiplantae as described previously [22]. Portions of seven nuclear genes (actin, GPI, GapA, OEE1, 40 S ribosomal protein S9 and 60 S ribosomal proteins L3 and L17) were amplified, cloned when necessary and sequenced as described in Cocquyt et al. [[22] and Additional file 1]. A histone H3 gene was amplified using the same PCR conditions with an annealing temperature of 55°C. The primers were based on a *Cladophora coelothrix *cDNA sequence aligned with GenBank sequences from green algae and land plants (His-F: 5'-ATG GCI CGT ACI AAG CAR AC-3' and His-R: 5'-GGC ATG ATG GTS ACS CGC TT-3'). In addition, total RNA was extracted from *Ignatius tetrasporus *and the bryopsidalean species *Caulerpa *cf. *racemosa *as described previously [21]. Portions of actin, β-tubulin, and HSP90 genes including the stop codon were amplified from these taxa by 3' RACE using the First Choice RLM-RACE kit (Ambion) using nested degenerate primers (actin-outer: 5'-TAC GAA GGA TAC GCA CTN CCN-3' C and actin-inner: 5'- GAG ATC GTG CGN GAY ATH AAR GA-3'; β-tubulin-outer: 5'-GAT AAC GAG GCT CTN TAY GAY ATH TG-3' and β-tubulin-inner: 5'-CCT TTC CGA CGG CTN CAY TTY TT-3'; HSP90-outer: 5'-ATG GTC GAT CCN ATH GAY GAR TA-3' and HSP90-inner: 5'-GCT AAG ATG GAG MGN ATH ATG AA-3'). ). These sequences were deposited in Genbank under accession numbers HQ332547-HQ332551.

### Genetic codes

The presence of a non-canonical nuclear genetic code in green algal taxa was detected by screening alignments of nuclear genes for supposed stop codons at positions coding for glutamine in other green plant taxa and by the presence of only TGA as a functional stop codon at the predicted 3' end of genes. The presence of the standard code was inferred if only canonical glutamine codons were present and all three stop codons occurred at the predicted end of genes.

### Molecular phylogenetics

We constructed a reference phylogeny of the Viridiplantae based on a 10-gene alignment to study the phylogenetic distribution of the standard and non-canonical genetic code [see Additional file 2]. For a detailed treatment of the methods used for tree reconstruction we refer to Cocquyt et al. [22]; we will only provide a summary here. The phylogenetic analysis was carried out on an alignment consisting of the seven nuclear genes mentioned above, together with SSU nrDNA and the plastid genes *rbc*L and *atp*B. Histone genes were excluded from the analysis because they are known to be duplicated across genomes [42,43]. Phylogenetic analyses were carried out with model-based techniques (maximum likelihood and Bayesian inference) after selection of a suitable partitioning strategy and models of sequence evolution. The model selection procedure proposed 8 partitions: SSU nrDNA was partitioned into loops and stems (2 partitions) and nuclear and plastid genes were partitioned into codon positions (2 × 3 partitions). GTR+Γ_8 _was the preferred model for all partitions. Noise-reduction techniques were applied to counteract the erosion of ancient phylogenetic signal caused by fast-evolving sites. The phylogenetic tree presented here is based on the 75% slowest-evolving sites [22].

### Evolution of glutamine codon usage

The evolution of glutamine codon usage was estimated using ancestral state inference techniques. The frequency of the two canonical and two non-canonical glutamine codons was calculated for each species in the phylogenetic tree. Codon frequencies were mapped along the reference tree using the ace function of the APE package [44]. This function estimates ancestral character states by maximum likelihood optimization [45]. The branch lengths were based on ML estimates because we consider them to be a more relevant approximation of the amount of codon usage evolution that can be expected to take place than absolute time [cf. [23]]. The output from APE was mapped onto the reference tree with TreeGradients v1.03 [46] to plot ancestral states as colors along a color gradient.

### Topological hypothesis testing

Approximately unbiased tests [AU test, [47]] were used to test an alternative relationship between ulvophycean orders as suggested by the distribution of the canonical genetic code (see results). Site-specific likelihoods were calculated by maximum likelihood optimization in Treefinder using the same model specifications as for phylogenetic inference [22]. AU tests were performed with CONSEL V0.1i [48].

## Abbreviations

eRF1: eukaryotic release factor 1; BV: boostrap value; CAR: CAG or CAA; GapA: glyceraldehyde-3-phosphate dehydrogenase; GPI: glucose-6-phosphate isomerase; Gln: glutamine; HSP: heat shock protein; Lys: lysine; ML: maximum likelihood; OEE1: oxygen-evolving enhancer protein; PP: posterior probability; Ser: serine, TAR: TAG or TAA.

## Authors' contributions

EC, ODC and FL designed the study. EC and GHG carried out lab work. EC and FL maintained algal cultures. EC and HV analyzed data. EC and ODC drafted the manuscript and FL, HV, PJK and GHG contributed to the interpretation of the data. All authors revised and approved the final manuscript.

## Supplementary Material

Additional file 1 **GenBank accession numbers**. Table with the Genbank accession numbers. Click here for file

Additional file 2 **Alignment**. nexus file of the data matrix containing 7 nuclear genes, SSU nrDNA and plastid genes *rbc*L and *atp*B. Click here for file
