# Supplementary material for: Complex phylogenetic distribution of a non-canonical genetic code in green algae
Source: BMC Evol Biol. 2010 Oct 26;10:327. doi: 10.1186/1471-2148-10-327 (PMC2984419; doi:10.1186/1471-2148-10-327)
Supplement: Additional file 1 — GenBank accession numbers. Table with the Genbank accession numbers. [file 1471-2148-10-327-S1.PDF]

Additional table 1. Genbank accession numbers for nucleotide sequences of actin, glucose-6-phosphate isomerase, glyceraldehydes-3-phosphate dehydrogenase, histone, oxygen evolving protein, 40S ribosomal protein S9 and 60S ribosomal protein L3 and L17. Sequences generated in Cocquyt et al. (2010, MBE 2010, 9: 2052-2061) are in bold and sequences generated for this study are marked in bold with an asterisk.

	actin	GPI	GapA	OEE1	histone	RP 40S S9	RP 60S L3	RP 60S L17
**Chlorophyta**								
**Ulvophyceae**								
**Ulvales - Ulotrichales**								
Acrochaete repens		**GQ421554**	**GQ421577**	**GQ421488**	**HQ336942***	**GQ421511**		
Bolbocoleon piliferum		**GQ421557**	**GQ421579**		**HQ336945***			
Halochlorococcum spp	**GQ421548**	**GQ421567**			**HQ336957***			
Ulva intestinalis	**GQ421553**	**GQ421575**	**GQ421592**	**GQ421509**	**HQ336969***	**GQ421519**		
Ulva sp.	AB106563 (*U. pertusa*)	**GQ421574**	**GQ421591**	**GQ421508**	**HQ336970***			
**Ignatius**								
Ignatius tetrasporus			**GQ421587**	**GQ421498**	**HQ336958***			**GQ421534**
**Bryopsidales**								
Bryopsis sp.			**GQ421582**	**GQ421492**/ AB293980 (*B. plumosa*)	**HQ336947***		**GQ421520**	
Codium sp.			**GQ421585**	**GQ421495**	**HQ336952***			**GQ421533**
Derbesia sp.		**GQ421565**	**GQ421586**	**GQ421496**	**HQ336954***			
Halimeda spp		**GQ421566** (H. cuneata2)			**HQ336956***			
**Dasycladales**								
Acetabularia acetabulum	**GQ421542**	AAL00001487 (TBestDB)		**GQ421487**	**HQ336941***		EC096877	CF259099
Bornetella sphaerica			**GQ421581**	**GQ421491**	**HQ336946***			
**Blastophysa**								
Blastophysa rhizopus		**GQ421555**	**GQ421578**	**GQ421489**	**HQ336943***			
**Cladophorales**								
Boergesenia forbesii	**GQ421543**	**GQ421556**			**HQ336944***	**GQ421512**		**GQ421528**
Boodlea composita	**GQ421544**	**GQ421558**	**GQ421580**	**GQ421490**		**GQ421513**		**GQ421529**
Cladophora albida		**GQ421562**	**GQ421584**		**HQ336950***			**GQ421531**
Cladophora coelothrix	**GQ421546**	**GQ421563**		**GQ421494**	**HQ336951***	**GQ421515**	**GQ421522**	**GQ421532**
Phyllodictyon spp	**GQ421549**	**GQ421571**		**GQ421503**	**HQ336964***	**GQ421517**	**GQ421527**	**GQ421537**
Siphonocladus tropicus	**GQ421551**	**GQ421572**		**GQ421505**	**HQ336966***	**GQ421518**		**GQ421539**
Valonia utricularis		**GQ421576**		**GQ421510**	**HQ336971***			**GQ421541**
**Trentepohliales**								
Trentepohlia aurea				**GQ421507**	**HQ336968***			
**Chlorophyceae**								
Chlamydomonas reinhardtii	D50838	**GQ421559**	L27668	X13826	**HQ336948***	**GQ421514**	**GQ421521**	XM_001693402
Scenedesmus obliquus	**GQ421550**	SOL00005809 (TBestDB)		**GQ421504**	**HQ336965***	EC189050	EC189501	**GQ421538**
Volvox carteri	M33963			AF110780		FD918100	FD837306	
**Trebouxiophyceae**								
Parachlorella kessleri	**GQ421545**	**GQ421560**	**GQ421583**	**GQ421493**	**HQ336963***			**GQ421530**

	actin	GPI	GapA	OEE1	histone	RP 40S S9	RP 60S L3	RP 60S L17
Helicosporidium sp.	AF317896					CX128902		CX128917
Prototheca wickerhamii	EC181529					EC181191	EC183246	EC180670
**Prasinophyceae**								
**Chlorodendrales**								
Scherffelia dubia	AF061018		DQ270259	AJ919716		AJ919712	AL132935	AJ919390
Tetraselmis spp	**GQ421552** (*T. striata*)	**GQ421573** (*T. striata*)		**GQ421506**/ AB293977 (*T. cordiformis*)	**HQ336967***			**GQ421540** (*T. striata*)
**Mamiellales**								
Ostreococcus tauri	CR954216	**GQ421570**	DQ649076	**GQ421502**	**HQ336962***		**GQ421526**	CR954206
**Pseudoscourfieldiales**								
Nephroselmis olivacea	EC732532	**GQ421569**	**GQ421590**	**GQ421501**/ AB293978	**HQ336961***			
**Streptophyta**								
**Mesostigmatales**								
Mesostigma viride	AF061020	**GQ421568**	**GQ421589**	**GQ421500**/ DN255652	**HQ336960***		**GQ421525**	**GQ421536**
**Chlorokybales**								
Chlorokybus atmophyticus		**GQ421561**	DQ270263		**HQ336949***			
**Klebsormidiales**								
Entransia fimbriata				**GQ421497**	**HQ336955***		**GQ421523**	
Klebsormidium flaccidum			**GQ421588**	**GQ421499**	**HQ336959***	**GQ421516**	**GQ421524**	**GQ421535**
**Zygnematales**								
Closterium sp.	**GQ421547**	**GQ421564**		AB293981				
Spirogyra sp.	AF061021		AJ246030					
**Coleochaetales**								
Coleochaete scutata	AF061019		DQ270264		**HQ336953***			
**Charales**								
Chara spp	DQ846905 (*C. contraria*)		DQ270262 (*C. vulgaris*)	AB293979 (*C. braunii*)				
**Embryophyta (land plants)**								
**Marchantiophyta (Liverworts)**								
Marchantia polymorpha	AB100427	BJ863864	AJ246026	BJ844290		BJ861755	BJ852563	BJ853031
**Bryophyta (Mosses)**								
Physcomitrella patens	XM_001782636	XM_001760154	DQ270266	XM_001763206		XM_001754317	XM_001782386	XM_001767807
**Spermatophyta (Seed plants)**								
Arabidopsis thaliana	M20016	AB044951	NM_101161	AJ145957		AB010077	M32655	AC004393
Oryza sativa	X16280	D45217	NM_001059519	NM_001049669		NM_001055539	D12630	NM_001069236
**other eukaryotes**								
**Archaeplastida**								
**Glaucocystophyta**								
Cyanophora paradoxa	CPU90325	DQ812897	DQ270258	AJ784854		EC666063	EC661414	EC666069
**Rhodophyta**								
**Cyanidiophyceae**								
Cyanidioschyzon merolae	D32140	AP006502	AP006492	AB159597			AP006495	
